# Machine learning-based identification of core regulatory genes in hepatocellular carcinoma: insights from lactylation modification and liver regeneration-related genes

**DOI:** 10.3389/fonc.2025.1683704

**Published:** 2025-11-14

**Authors:** Yu Yang, Yiwei Hou, Li Yi, Chongyuan Chen, Xiang Li, Yashan Wang, Yunxi Fu, Mingzheng Hu, Rongchun Xing

**Affiliations:** 1 The First College of Clinical Medical Science, China Three Gorges University, Yichang, Hubei, China; 2 Department of Hepatobiliary Surgery, Yichang Central People’s Hospital, Yichang, Hubei, China; 3 Department of Endocrinology, Yichang Central People’s Hospital, Yichang, Hubei, China; 4 Medical Technology College of Qiqihar Medical College, Qiqihar, Heilongjiang, China

**Keywords:** lactylation, liver regeneration, hepatocellular carcinoma, machine learning, bioinformatics analysis

## Abstract

**Introduction:**

Hepatocellular carcinoma (HCC) progression shares metabolic-epigenetic features with physiological liver regeneration, yet the regulatory interplay remains poorly defined. We hypothesize that lactylation, a novel post-translational modification, serves as a key nexus linking these processes.

**Methods:**

We integrated lactylation modification profiles with transcriptomic data from three murine liver regeneration datasets (GSE20426, GSE70593, GSE4528). Machine learning algorithms, including LASSO regression and SVM-RFE, were employed to prioritize core regulatory genes. Functional characterization involved enrichment, immune infiltration, and correlation analyses. The prognostic and diagnostic value of the identified genes was validated in HCC cohorts, and their overexpression was confirmed in clinical HCC specimens using qPCR and Western blot.

**Results:**

Multi-omics analysis revealed 793 differentially expressed genes during liver regeneration, with 18 overlapping lactylation-related candidates. Machine learning prioritized six core genes (Ccna2, Csrp2, Ilf2, Kif2c, Racgap1, Vars) enriched in cell cycle regulation and DNA repair pathways. These genes demonstrated a strong correlation with immune microenvironment remodelling, particularly CD8^+^ T cells and M1 macrophages. Prognostic validation in HCC cohorts revealed significant overexpression of these genes in tumours, with elevated Kif2c and Ccna2 predicting poor survival. Crucially, Csrp2 exhibited superior diagnostic efficacy (AUC > 0.8) compared to conventional biomarkers. Experimental validation via qPCR and Western blot confirmed marked upregulation of all six genes at both mRNA and protein levels in clinical HCC specimens (p < 0.0001).

**Discussion:**

This work uniquely establishes lactylation as a metabolic-epigenetic bridge linking physiological regenerative pathways to oncogenesis. By leveraging liver regeneration models and machine learning, we propose the identified gene panel as dual-purpose biomarkers for HCC diagnosis and therapeutic targeting, offering new insights into the metabolic-epigenetic regulation of HCC.

## 1 Introduction

Liver regeneration represents a paradigmatic model of finely regulated cellular proliferation, which is associated with the characteristic uncontrolled growth seen in HCC. These two processes may involve overlapping proliferative mechanisms, yet the key molecular determinants of physiological repair versus malignant transformation remain unclear (1). Recent studies suggest that metabolic-epigenetic interactions, particularly through lactylation, a novel post-translational modification, play a central role in regulating hepatocyte fate decisions (2).

The liver’s unique regenerative capacity has long been considered a classic model for studying controlled cell cycle processes (3, 4). After partial hepatectomy, quiescent hepatocytes initiate precise, time-dependent proliferation programs through metabolic reprogramming and epigenetic remodeling, enabling functional reconstruction within days (5). The transient activation of this proliferative pathway requires the precise coordination of energy metabolism and chromatin remodeling, with lactylation potentially serving as a critical component of this regulatory hub (6). Recent research has revealed that lactate-induced histone modifications guide regenerative gene programs by regulating chromatin accessibility (7). Interestingly, the tumor microenvironment can hijack similar lactylation-driven mechanisms to sustain oncogenic signaling (8), suggesting that lactylation may serve as a molecular regulator controlling proliferative checkpoints.

Notably, lactylation modifications in HCC are often abnormally hyperactivated, and this epigenetic regulation plays a key role in driving HCC progression (9). Lactylation has gradually emerged as a metabolically sensitive epigenetic mechanism linking glycolytic flux to chromatin state. Its molecular nature involves the covalent binding of lactate molecules to histone lysine residues, reshaping the transcriptional landscape under metabolic stress. Physiological processes such as liver regeneration demonstrate that lactylation modifies promoter regions to activate pro-regenerative pathways (6, 7). Conversely, cancer cells exploit similar mechanisms to stabilize oncogenic transcription factors and enhance chemoresistance. This functional duality positions lactylation at the critical interface between metabolic adaptation and epigenetic regulation, potentially governing the phenotypic switch between regeneration and carcinogenesis (10).

The role of lactylation in liver function remains poorly understood both domestically and internationally. While its involvement in macrophage polarization during liver injury and metabolic regulation in HCC models has been documented, the specific mechanisms by which this modification coordinates regeneration-specific gene networks are yet to be elucidated (11–13). The significance of this knowledge gap arises from the shared metabolic features of regenerative hepatocytes and malignant cells: both exhibit a Warburg-like effect, characterized by glycolysis, lactate accumulation, and proliferation-dependent epigenetic remodeling (14). We hypothesize that lactylation, as a conserved regulatory mechanism across physiological and pathological environments, determines divergent biological outcomes through its spatiotemporal-specific modification patterns. Therefore, genes co-regulated by lactylation in both physiological regeneration and tumor growth may constitute core drivers of HCC pathogenesis.

Current traditional methods for identifying oncogenes face significant limitations in exploring metabolic-epigenetic interactions. Conventional differential expression analysis often overlooks the environmental dependency of modification effects, while bulk sequencing technologies struggle to capture cell type-specific lactylation signatures (15). To overcome these limitations, we employ an integrated bioinformatics strategy that combines lactylome data with longitudinal regeneration transcriptomes. Notably, several groups have previously investigated the role of lactylation in HCC pathogenesis (16, 17), yet these studies rarely integrated physiological liver regeneration models (a paradigm of controlled hepatocyte proliferation) to distinguish “regenerative” vs. “oncogenic” lactylation effects. This omission is notable, as murine liver regeneration models have been widely validated to recapitulate the core molecular pathways of human hepatic repair—for instance, studies by Jian Zhao et al. (18) and Costanza Lamperti et al. (19) demonstrated that key cell cycle regulators (e.g., cyclins, CDKs) and metabolic reprogramming events in the murine partial hepatectomy (PHx) model are highly conserved in human liver regeneration after surgical resection, confirming the translational value of murine data for human liver biology research. To address this gap, we employ an integrated bioinformatics strategy that combines lactylome data with longitudinal regeneration transcriptomes, and further uses machine learning (LASSO + SVM-RFE) for gene prioritization. This approach circumvents the limitations of single-omics analyses and single-model designs, enabling precise pinpointing of modification-sensitive oncogenic nodes that bridge physiological regeneration and pathological carcinogenesis.

## 2 Methods

### 2.1 Data collection

**Figure 1:**
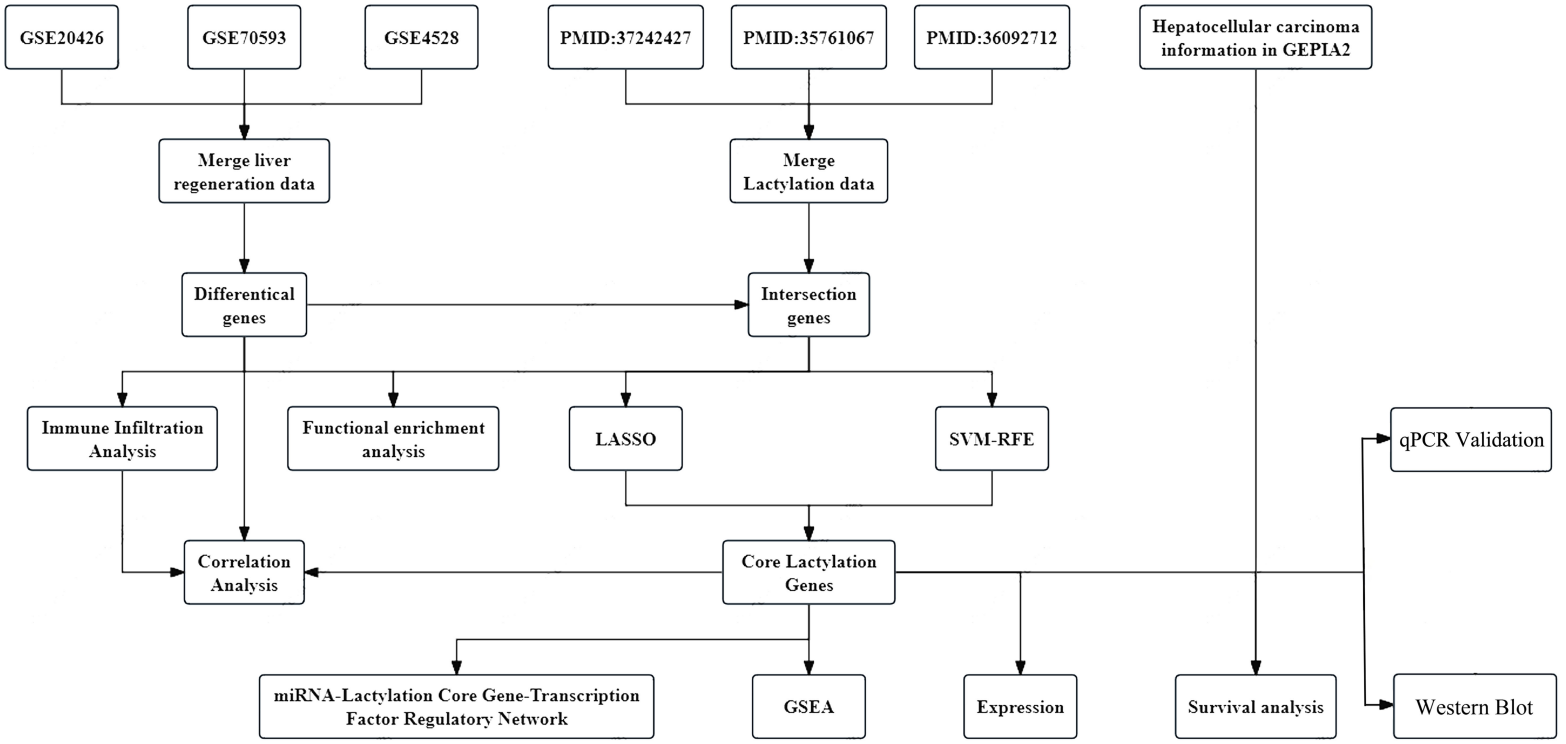
Study flow chart. The study investigates the molecular mechanisms of liver regeneration in mice by collecting and preprocessing gene expression datasets, performing differential gene expression screening and enrichment analysis, and integrating machine learning with immune infiltration analysis to identify and explore core lactylation-related genes. Regulatory network prediction, cancer expression profiling, and statistical validation were conducted to further elucidate their functional roles. Moreover, key findings were experimentally validated through molecular biology techniques, including quantitative PCR (qPCR) and Western blotting (WB).

The sequence of steps in our research methodology is depicted in the workflow diagram below, labelled as **Figure 1**. To further analyze the molecular mechanism of liver regeneration after PHx and strengthen the correlation between the “mouse regeneration model human HCC” in the original data system, we further clarified the selection logic and analysis basis of the original dataset to ensure the rigor of the research foundation:

To deeply understand the molecular mechanisms of liver regeneration after partial hepatectomy in mice, we selected three datasets (GSE20426, GSE70593, GSE4528) from the GEO database. Also, we consulted literature [PMID:37242427 (16), PMID:35761067 (17), PMID:36092712 (20)] to compile known lactylation gene information, ensuring the scientific and practical significance of the gene list used in the research.

### 2.2 Data preprocessing

A series of methods was used for data quality control. The “limma” and “sva” packages in R (v4.2.2) were applied to remove batch effects across the merged datasets. This approach of integrating multiple datasets followed by batch effect correction is an established strategy to increase the effective sample size and statistical power, particularly when individual cohort sizes are limited (21). Notably, this multi-dataset integration strategy for mouse liver data has been successfully applied to identify biomarkers for human HCC. For instance, Zeyu Wang et al. (22) integrated 4 mouse liver regeneration datasets and identified a 5-gene signature, which was later validated to predict recurrence in human HCC patients. This finding demonstrates the translational validity of the analytical framework. PCA, with “FactoMineR” and “factoextra” packages in R, was conducted to assess differential correction and visualize sample distribution. The “preprocessCore” package was used for quantile normalization and standardization to reduce the impact of outliers and ensure comparability across samples. Quantile normalization is a widely adopted and robust method for eliminating technical variations between arrays in gene expression studies, effectively aligning the distribution of probe intensities across all samples to a common target distribution (23, 24).

### 2.3 Differential gene, intersection gene screening and analysis

We used the “limma” package in R with strict criteria (|log fold change(logFC)| > 0.5, adj.P.Val < 0.05) to identify differentially expressed genes between normal/sham-operated and liver-regenerating groups after partial hepatectomy. The “Venn” package was then used to find the intersection of lactylation genes and differential genes. Volcano plots, heat maps, and box plots were created using “ggplot2” and “pheatmap” packages to analyze these genes.

### 2.4 Enrichment analysis

GO and KEGG analyses, performed with the “clusterProfiler” package in R on differential and intersection genes, revealed their involvement in biological processes, molecular functions, etc. Results were sorted by gene ratio, with circle size and color representing gene quantity and P-values.

### 2.5 Identification of core lactylation genes using machine learning

LASSO is performed using the glmnet function (alpha=1, corresponding to an L1 penalty) and undergoes 10-fold cross-validation via cv.glmnet (nfolds=10, type.measure=“deviance”), with lambda.min set as the feature selection threshold. The SVM-RFE method utilizes the caret::rfe function, applying svmRadial (Radial Basis Kernel, RBF) as the core. RBF is selected for its flexibility in capturing nonlinear boundaries. In the rfeControl setting, the method is set to “cv” (default 10% discount), which allows recursive feature elimination and automatically adjusts parameters (sigma and C) for each subset of data through caret. Given the priority of public datasets and the small sample size, the predictive performance of these genes is so strong that all values are 1. Further screened genes by iteratively removing those with less classification contribution. The gene sets from both methods were intersected to determine core lactylation genes. Correlation analyses and ROC curve analysis, with the “pROC” package, were conducted to evaluate these genes.

### 2.6 Immune infiltration analysis

Based on gene expression data, the R package CIBERSORT, a deconvolution-based tool, was used to estimate immune cell composition in liver regeneration samples (GSE20426, GSE70593, GSE4528). After inferring immune cell composition, the Wilcoxon rank-sum test was used to compare differences between control and experimental groups, with P < 0.05 considered significant. The application of CIBERSORT in mouse regeneration data is consistent with the previous work by Lea Lemaitre et al. (25). They used mouse liver immune infiltration patterns to identify macrophage-T cell crosstalk pathways, which were later confirmed to regulate immune evasion in human HCC. This finding supports the relevance of our mouse immune analysis to human diseases.

### 2.7 Correlation analysis between core lactylation genes, immune cells, and all genes

Spearman correlation analysis, using “ggplot2” and “ggstatsplot” packages in R, explored the relationship between core lactylation gene expression and immune cell infiltration. A correlation heatmap, created with the “pheatmap” package, showed the top 50 genes positively correlated with core lactylation genes.

### 2.8 Single gene GSEA analysis of core lactylation genes

Single-gene gene set enrichment analysis (GSEA), with the “clusterProfiler” package in R on six core lactylation genes, explored their enrichment in molecular functions and signaling pathways. Results with adjusted P-values < 0.05 and FDR q < 0.025 were considered significant, and bar graphs were used to show the top 20 findings.

### 2.9 Prediction of miRNA-lactylation core gene - transcription factor regulatory network

The RegNetwork database and Cytoscape software were used to identify and construct the miRNA-lactylation core gene-transcription factor regulatory network. The NetworkAnalyst platform and MiRTarBase v8.0 database were further used to validate and visualize the network, providing clues for upstream regulatory mechanism research.

### 2.10 Differential expression analysis and survival analysis of core lactylation genes in hepatocellular carcinoma

Using the GEPIA2 database, we compared core lactylation gene expression in hepatocellular carcinoma and normal liver tissues with t-tests. Kaplan-Meier survival curves were used to analyze the correlation between gene expression and patient prognosis, aiming to find new biomarkers. It is noteworthy that multivariate Cox regression analysis adjusting for clinical covariates (such as tumor stage, grade, age, and liver function parameters) could not be performed due to the lack of detailed clinical annotation in the utilized public databases (e.g., TCGA, GEO). This limitation is common in large-scale bioinformatic studies relying on publicly available datasets, where comprehensive clinical information is often incomplete or unavailable (26, 27). Similarly, a direct head-to-head comparison of the diagnostic accuracy of our identified biomarkers with serum alpha-fetoprotein (AFP) levels was not feasible, as gene expression datasets and serum biomarker datasets are typically derived from distinct patient cohorts with different sample types (tissue versus blood) (28, 29).

### 2.11 Clinical cohort for experimental validation

For the experimental validation, we analyzed a cohort comprising n = 10 primary HCC tumors paired with n = 10 patient-matched adjacent non-tumor liver tissues (collected ≥2 cm from the tumor margin) from Yichang Central People’s Hospital. Inclusion criteria were: (1) adults (≥18 years) with pathologically confirmed, treatment-naïve primary HCC; (2) curative-intent surgical resection with available tumor and adjacent non-tumor tissue; (3) adequate tissue quantity/quality for RNA and protein extraction; (4) availability of complete core clinical covariates; and (5) provision of written informed consent with IRB approval. Patients were excluded based on: (1) prior loco-regional or systemic anti-cancer therapy; (2) mixed histology (e.g., HCC–intrahepatic cholangiocarcinoma); (3) recurrent or metastatic disease at sampling; (4) inadequate biospecimen quality (RIN < 6.0 or >50% necrosis on pathology); (5) severe acute hepatitis or active systemic infection at surgery; or (6) incomplete metadata. Regarding specimen handling, tissues were snap-frozen in liquid nitrogen within 30 minutes of resection and stored at −80°C. Total RNA was extracted using TRIzol reagent with chloroform phase separation, followed by DNase treatment and quality control (Nanodrop 260/280 ratio, Bioanalyzer RIN). Protein lysates were prepared in RIPA buffer supplemented with protease and phosphatase inhibitors, with concentrations determined by BCA assay. For qPCR, reactions were performed in technical triplicates using GAPDH as the internal control, and data were analyzed via the ΔΔCt method. Western blot signals were normalized to GAPDH, and band densitometry was quantified using ImageJ by analysts blinded to the clinical groupings. Normalization and batch handling procedures were as follows: for qPCR, Ct values were normalized to GAPDH and reported as ΔΔCt relative to the matched non-tumor tissue; for any RNA-seq-based validation, expression was normalized as TPM (or CPM) with batch effects adjusted using ComBat from the sva package; and for Western blotting, protein loading was standardized to 30 µg per lane with identical exposure settings across all membranes.

### 2.12 Validation of core lactylation genes in normal liver tissues and hepatocellular carcinoma by qPCR

qPCR was performed on 10 normal and 10 hepatocellular carcinoma tissue samples to validate lactylation modification genes. GAPDH was used as an internal control. RNA extraction followed a standard protocol, and cDNA synthesis was done with a commercial kit. qPCR was carried out with specific primers, and relative expression levels were calculated by the 2^(-ΔΔCt) method (**Table 1**).

**Table 1:** Primer sequences for q-PCR assay.

Gene	Forward primer (5′–3′)	Reverse primer (5′–3′)	Product size (bp)
CCNA2	TGGAAAGCAAACAGTAAACAGCC	GGGCATCTTCACGCTCTATTT	109
CSRP2	TGGGAGGACCGTGTACCAC	CCGTAGCCTTTTGGCCCATA	177
ILF2	GGGGAACAAAGTCGTGGAAAG	CCAGTTTCGTTGGTCAGCA	75
KIF2C	CTGTTTCCCGGTCTCGCTATC	AGAAGCTGTAAGAGTTCTGGGT	185
RACGAP1	TGCACGTAATCAGGTGGATGT	TGAATCTGTCGTTCCAGCTTTT	81
VARS	CGACTAGCAGGACTCCCTTTC	CGGCGTAACTGACCCACTG	174
GAPDH	GGAGCGAGATCCCTCCAAAAT	GGCTGTTGTCATACTTCTCATGG	197

This table presents the primer sequences used for the qPCR assay in the study. The sequences are provided for six core lactylation modification genes (CCNA2, CSRP2, ILF2, KIF2C, RACGAP1, VARS) along with the internal control gene GAPDH. Each row shows the forward and reverse primer sequences, as well as the product size in base pairs. These primer sequences were utilized to validate the expression levels of the lactylation-related genes in normal liver and hepatocellular carcinoma tissues, contributing to the experimental validation of the study’s findings.

### 2.13 Western blot analysis of protein expression in hepatocellular carcinoma and paired adjacent tissues

Western blotting was performed on hepatocellular carcinoma and adjacent tissues homogenized in RIPA buffer with protease inhibitors. Protein concentration was determined by BCA assay. Equalized samples were denatured, separated by SDS-PAGE, and electrotransferred to PVDF membranes. After blocking, membranes were incubated sequentially with primary and secondary antibodies, washed with TBST, and detected by chemiluminescence.

### 2.14 Statistical analysis

Count variable data were expressed as mean ± SD. One-way ANOVA (for ≥ 2 variables) or t-tests (for 2 variables) were used for count variable comparisons, and chi-square tests for categorical variables. Simple linear regression models were used for the correlation analysis between core lactylation genes and immune cells. All analyses were done with SPSS software, with P < 0.05 considered significant.

## 3 Results

### 3.1 Data quality control

**Figure 2:**
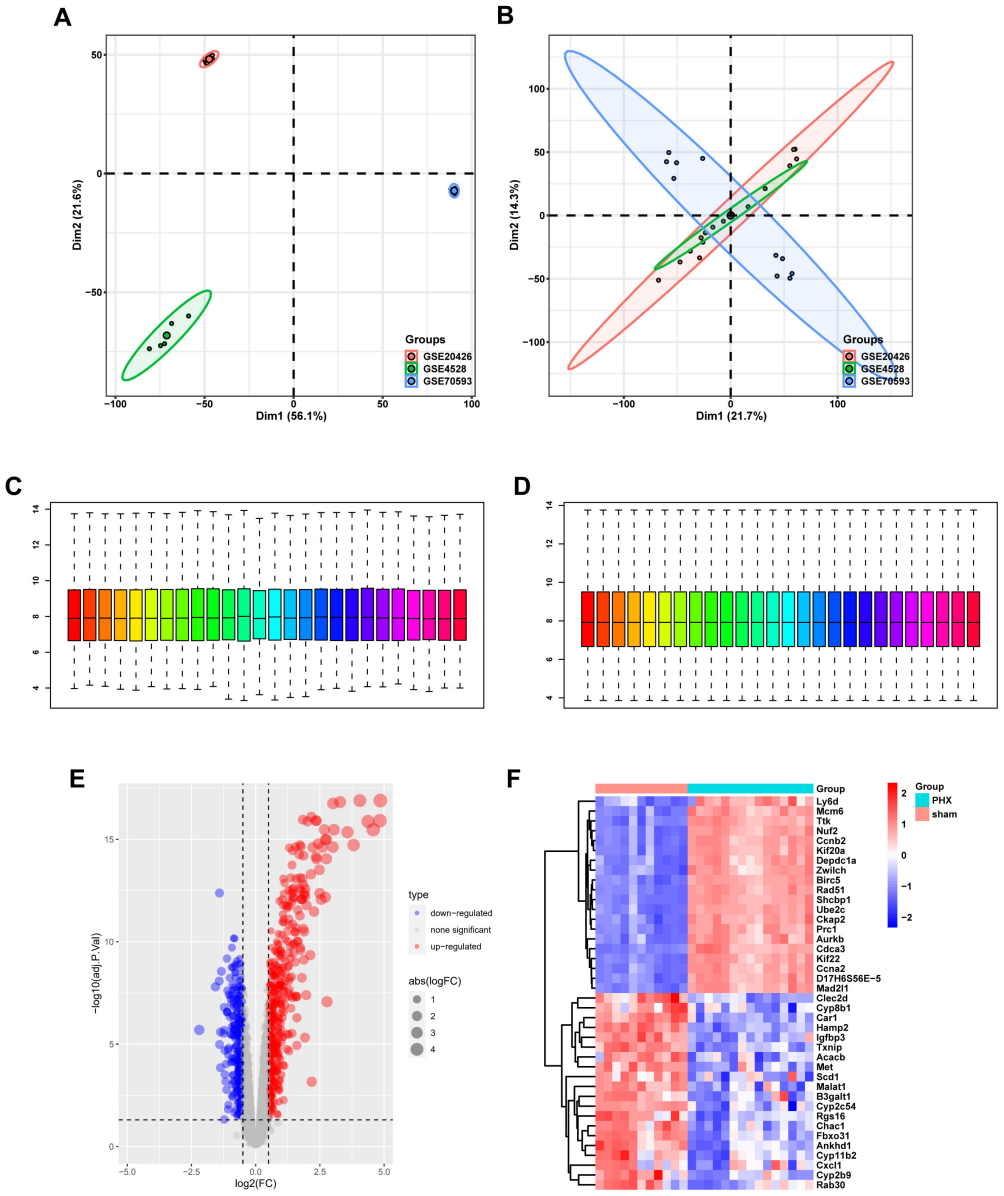
Gene expression profiling and normalization. **(A)** PCA of combined datasets prior to batch correction, showing high variability. **(B)** PCA after batch effect removal reveals improved clustering by biological factors. **(C)** Boxplot of gene expression levels before normalization, highlighting variability between samples. **(D)** Post-normalization boxplot showing uniform gene expression levels. **(E)** Volcano plot showcasing differentially expressed genes, with significant upregulation in red and downregulation in blue. **(F)** Heatmap displaying the expression of DEGs, where red indicates higher expression and blue indicates lower expression.

PCA and data normalization were carried out for datasets GSE20426, GSE70593, and GSE4528 (**Figures 2A, B**). With 26 mouse samples (11 in the control group and 15 in the PHx group), individual dataset analysis showed differences. But after merging, 26 samples and 9,224 genes overlapped, validating data similarity. Box plots before and after normalization demonstrated consistent medians, ensuring data quality for further analysis (**Figures 2C, D**).

### 3.2 Differential gene expression analysis

**Figure 3:**
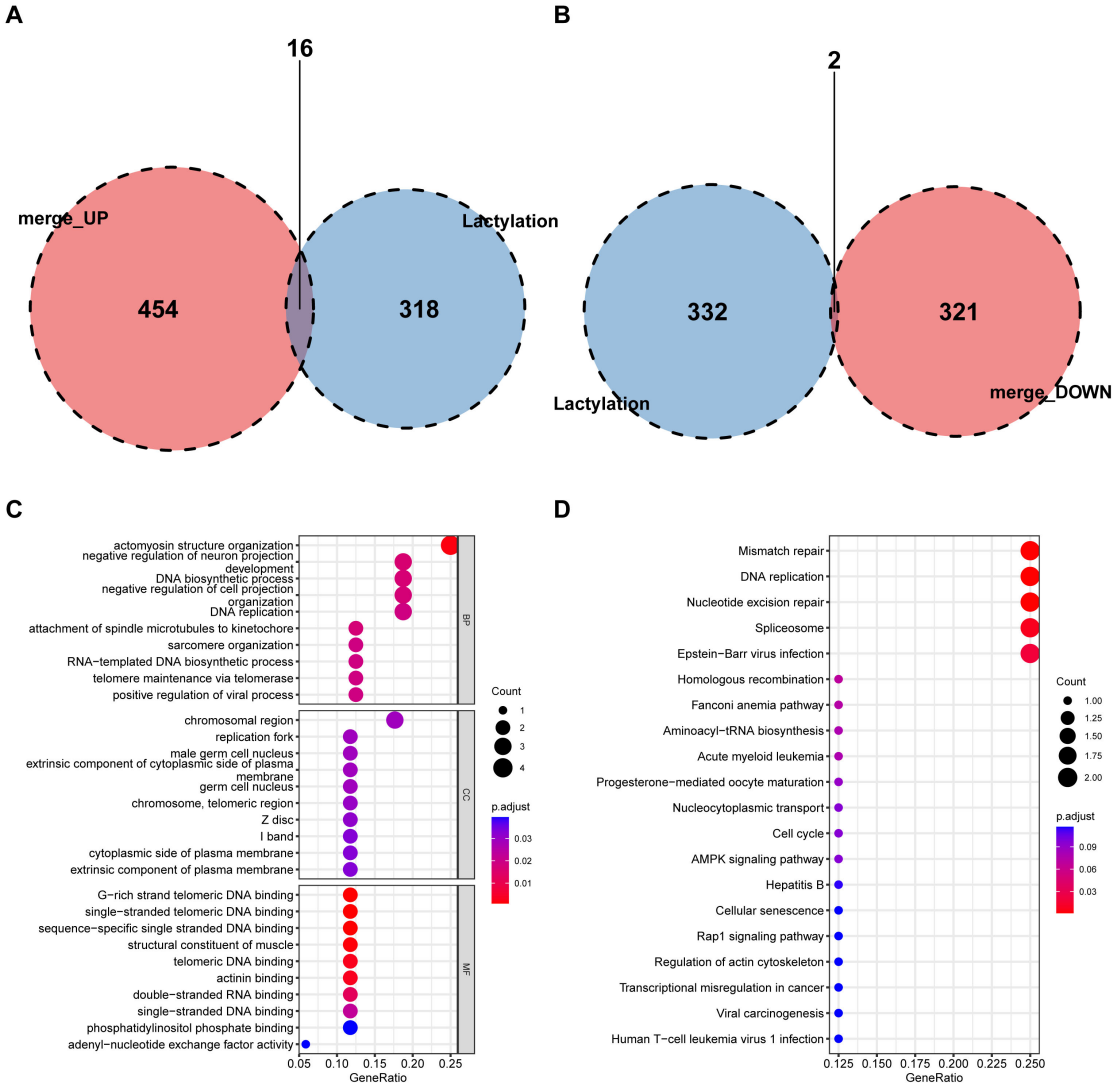
Intersection of DEGs with lactylation modification genes. **(A)** A Venn diagram shows the intersection of upregulated DEGs with lactylation modification genes, identifying 16 shared genes. **(B)** Downregulated DEGs intersecting with lactylation genes, revealing 2 shared genes. **(C)** GO enrichment analysis of shared genes in BP, CC, and MF categories. **(D)** KEGG pathway analysis highlights key metabolic and signaling pathways affected by intersected genes.

**Figure 4:**
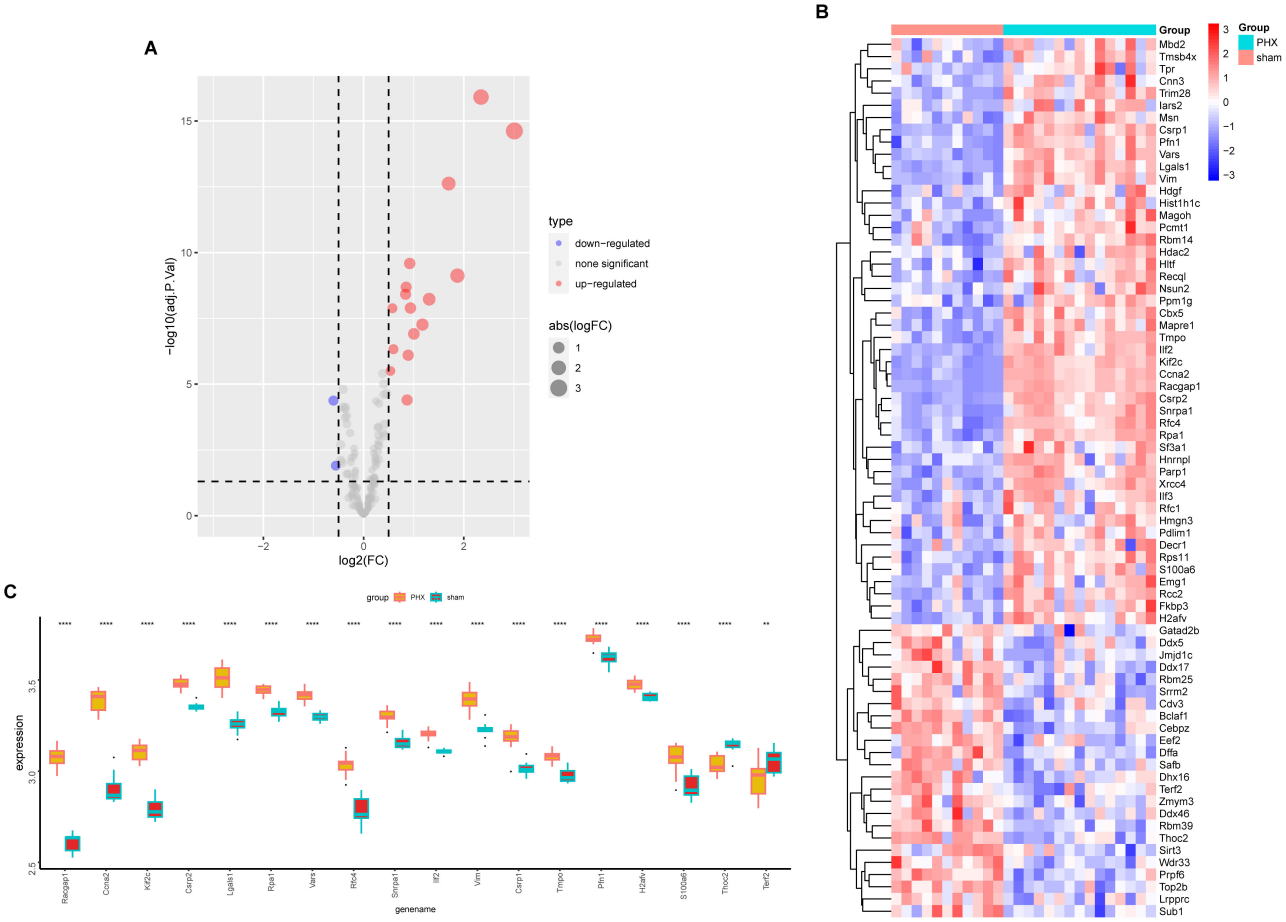
Lactylation modification gene expression analysis. **(A)** The volcano plot illustrates differential expression of lactylation modification genes between partial hepatectomy and control groups. **(B)** Heatmap displays expression profiles of lactylation-related genes across samples. **(C)** Boxplot shows expression differences of 18 intersected genes between groups; statistical significance is marked by asterisks (**p < 0.01, ****p < 0.0001).

Volcano and heat maps presented differentially expressed genes in liver regeneration. There were 470 upregulated and 323 downregulated genes (**Figures 2E, F**). For example, Ly6d and Mcm6 were highly expressed in the regeneration group, while Clec2d and Cyp8b1 were low. Intersection analysis of lactylation modification genes found 18 genes (16 upregulated and 2 downregulated) (**Figures 3A, B**). Racgap1 and Ccna2 were highly expressed among the upregulated ones, and Thoc2 and Terf2 were downregulated (**Figures 4A–C**).

### 3.3 Enrichment analysis

**Figure 5:**
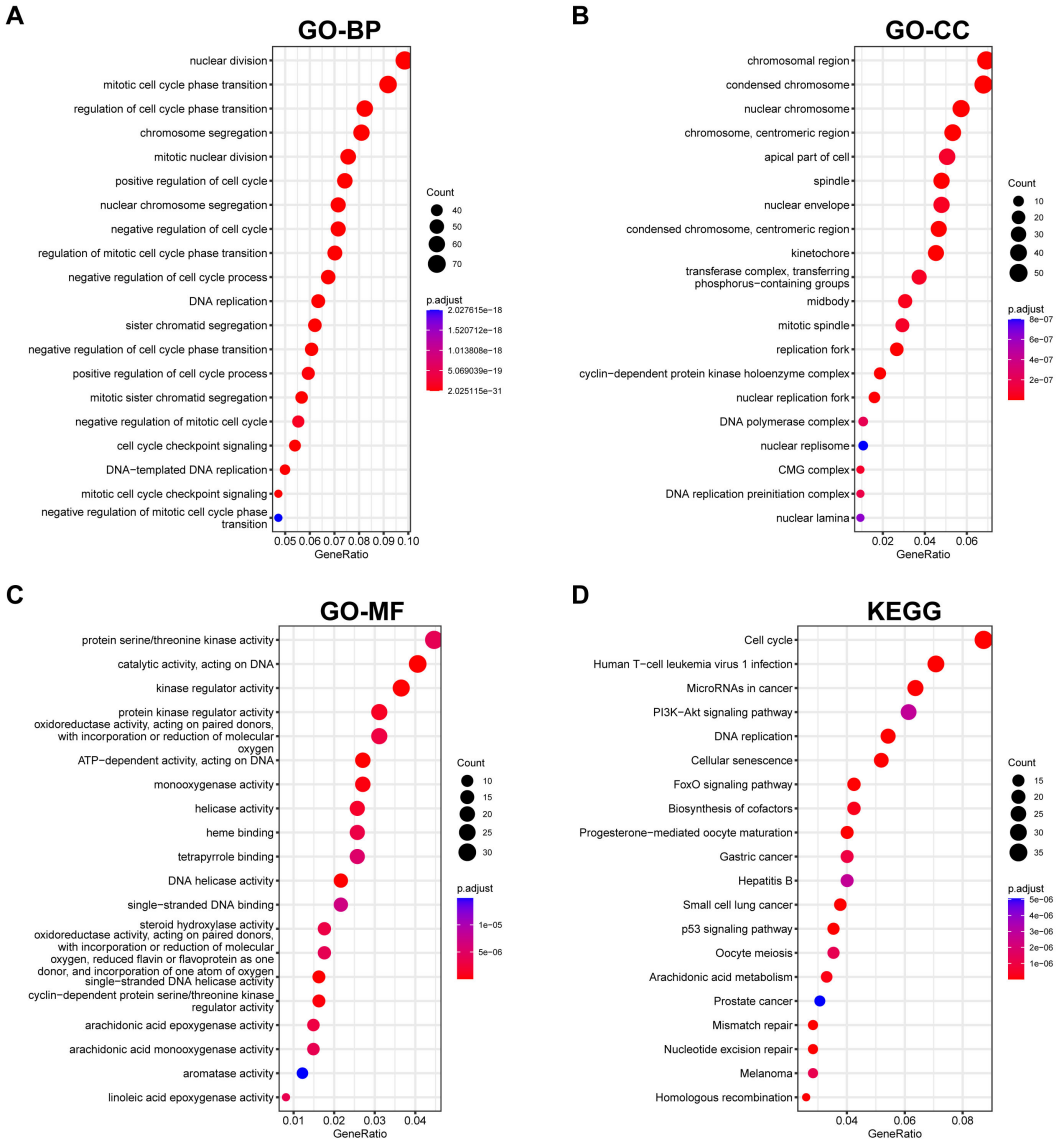
Functional enrichment analysis of DEGs. **(A–C)** GO enrichment analysis presents biological processes, cellular components, and molecular functions impacted by DEGs. **(D)** KEGG pathway analysis reveals pathways significantly influenced by DEGs, providing insights into potential biological mechanisms.

GO and KEGG pathway analyses were conducted on differential and intersecting genes. Differential genes were enriched in nuclear division, mitotic cell cycle, etc. in GO (**Figure 5A–C**) and Cell cycle and Human T-cell leukemia virus 1 infection in KEGG (**Figure 5D**). Lactylation modification genes were enriched in actomyosin structure organization in GO and Mismatch repair, DNA replication, etc. in KEGG (**Figures 3C, D**).

### 3.4 Screening of core lactylation modification genes

**Figure 6:**
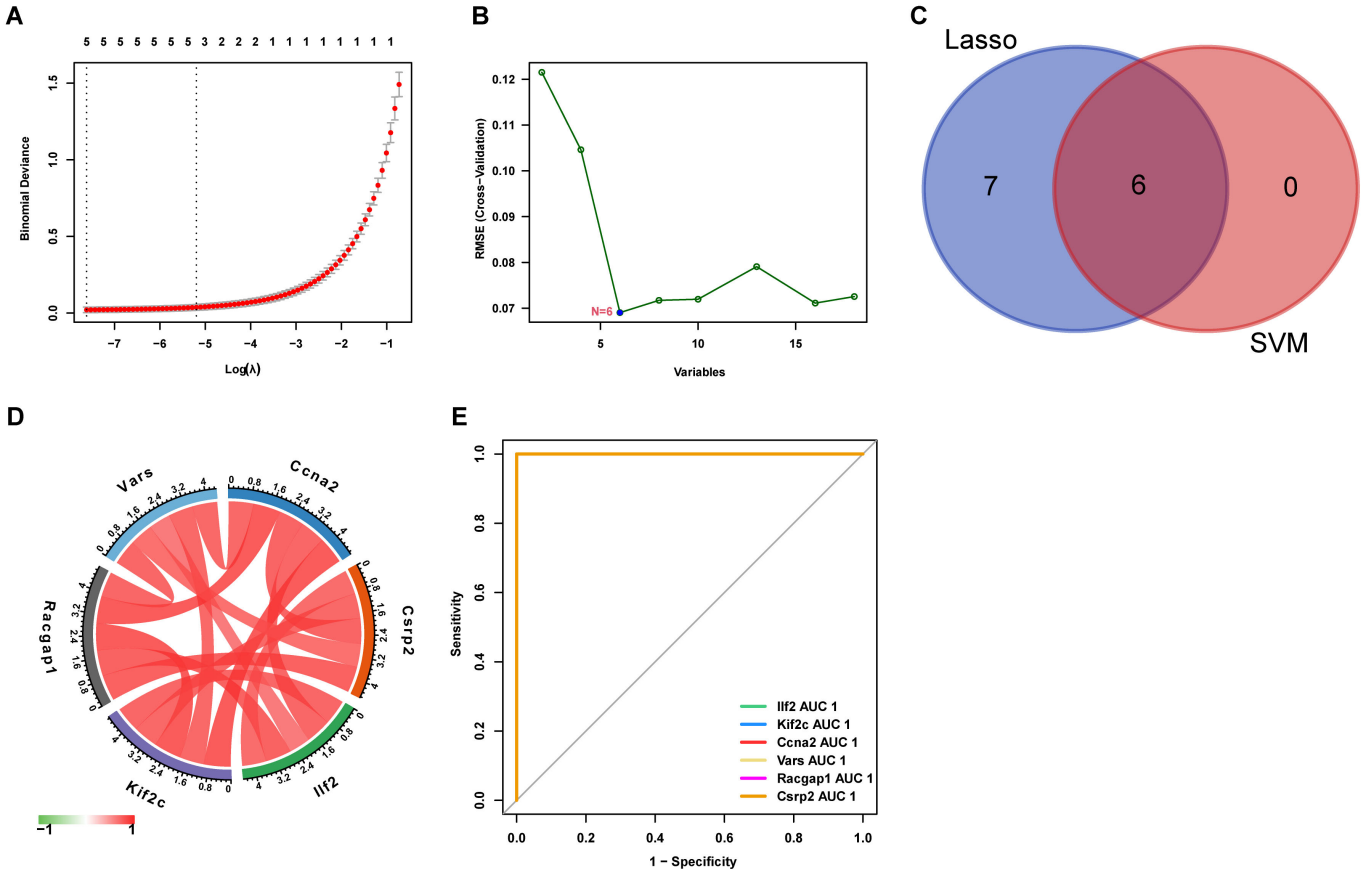
Feature selection for key genes using machine learning **(A)** LASSO regression selects 13 key genes from lactylation-related DEGs. **(B)** SVM identifies 6 core genes. **(C)** The intersection yielded six core genes **(D)** The correlation matrix shows relationships between these genes, with red indicating positive and green indicating negative correlation. **(E)** The ROC curve displays the predictive performance of the selected genes.

LASSO regression and SVM - RFE algorithms identified six core lactylation modification genes: Ccna2, Csrp2, Ilf2, Kif2c, Racgap1, and Vars, which were strongly correlated (**Figures 6A–D**). ROC curve analysis showed their strong predictive efficacy, especially Csrp2 with an AUC > 0.8 (**Figure 6E**).

### 3.5 Immune cell infiltration analysis

**Figure 7:**
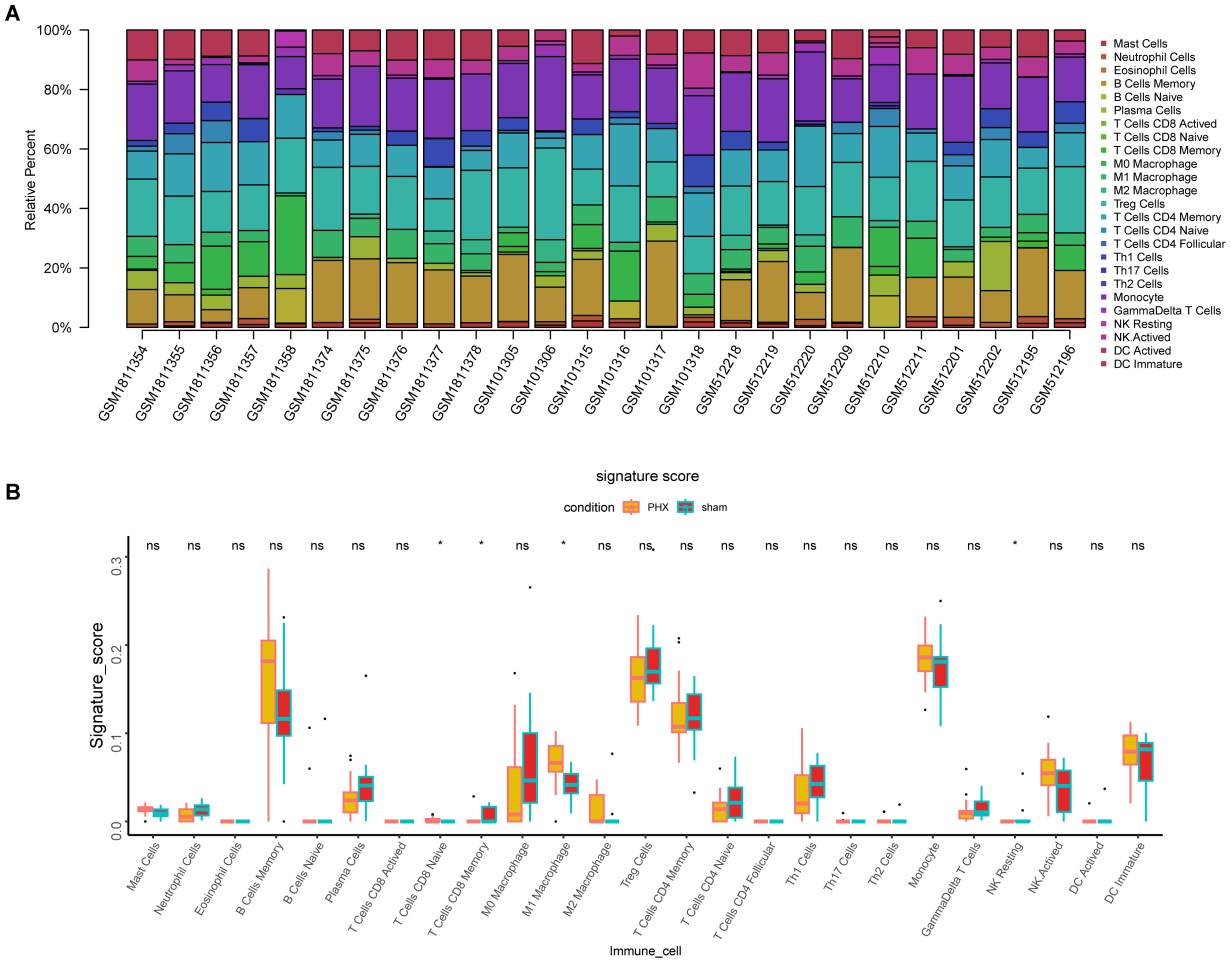
Immune cell infiltration analysis in partial hepatectomy samples. **(A)** Bar plot depicts immune cell type proportions in both partial hepatectomy and control samples. **(B)** Boxplot highlights significant differences in immune cell infiltration between these groups (*p < 0.05, ns, not significant).

Analysis of 25 immune cell types’ infiltration levels revealed significant differences between the liver regeneration and control groups for four immune cell types (P < 0.05) (**Figures 7A, B**). CD8+ naive T cells and M1 macrophages were highly expressed in the regeneration group, while CD8+ memory T cells and resting NK cells were low, indicating their roles in immune regulation during liver regeneration.

### 3.6 Correlation between core lactylation modification genes and immune infiltration

**Figure 8:**
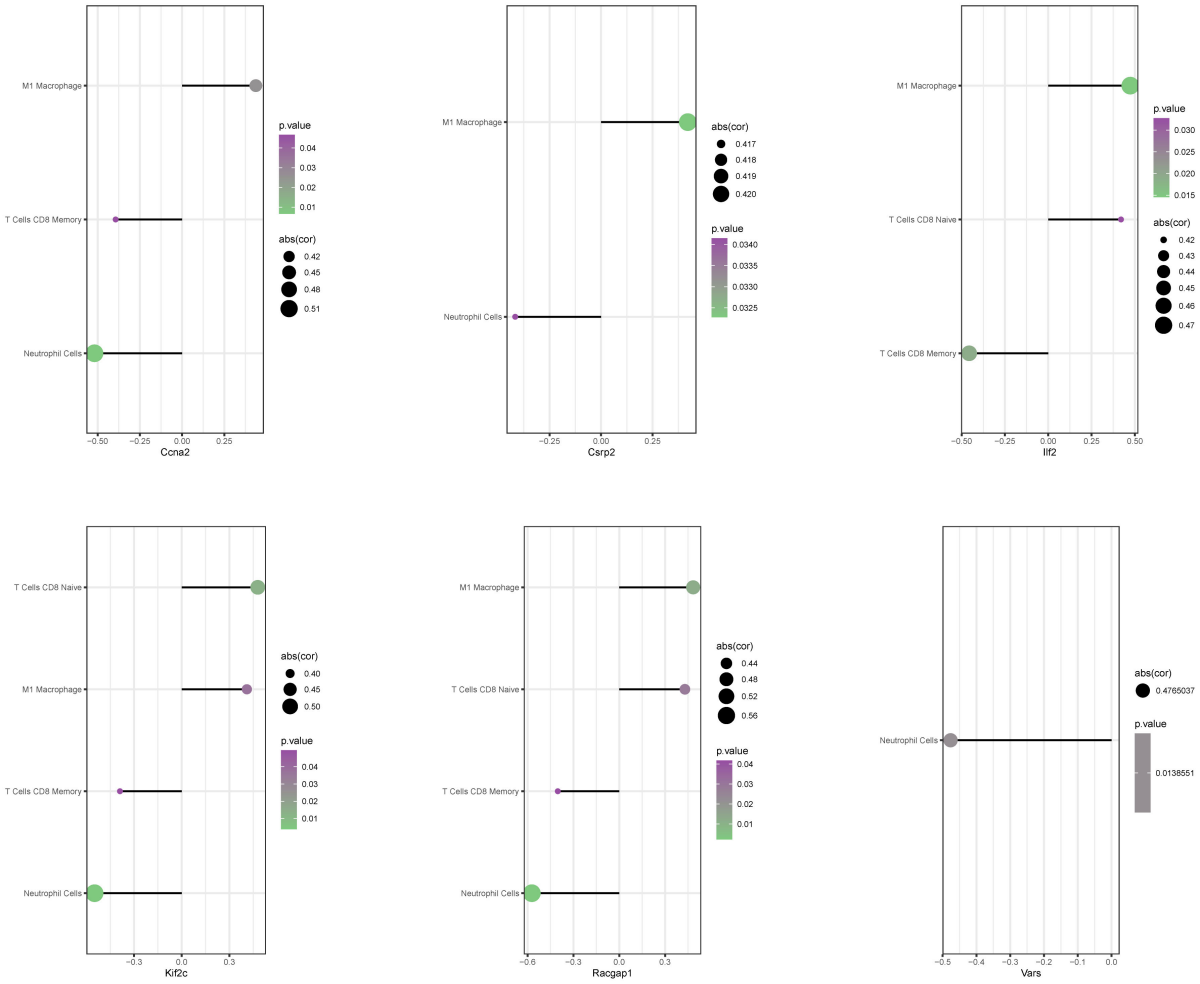
Correlation between core genes and immune cell infiltration. A bubble chart presents the correlation between 6 core genes and immune cell infiltration levels. The size of the circles represents correlation strength, while color intensity indicates p-values.

CIBERSORT algorithm analysis showed significant correlations. For example, Ccna2 was positively correlated with M1 macrophage infiltration but negatively with CD8 memory T cells and resting NK cells (**Figure 8**). Other genes also showed similar positive and negative correlations, suggesting that core lactylation modification genes can influence the immune microenvironment of liver regeneration.

### 3.7 Correlation between core lactylation modification genes and liver regeneration genes

**Figure 9:**
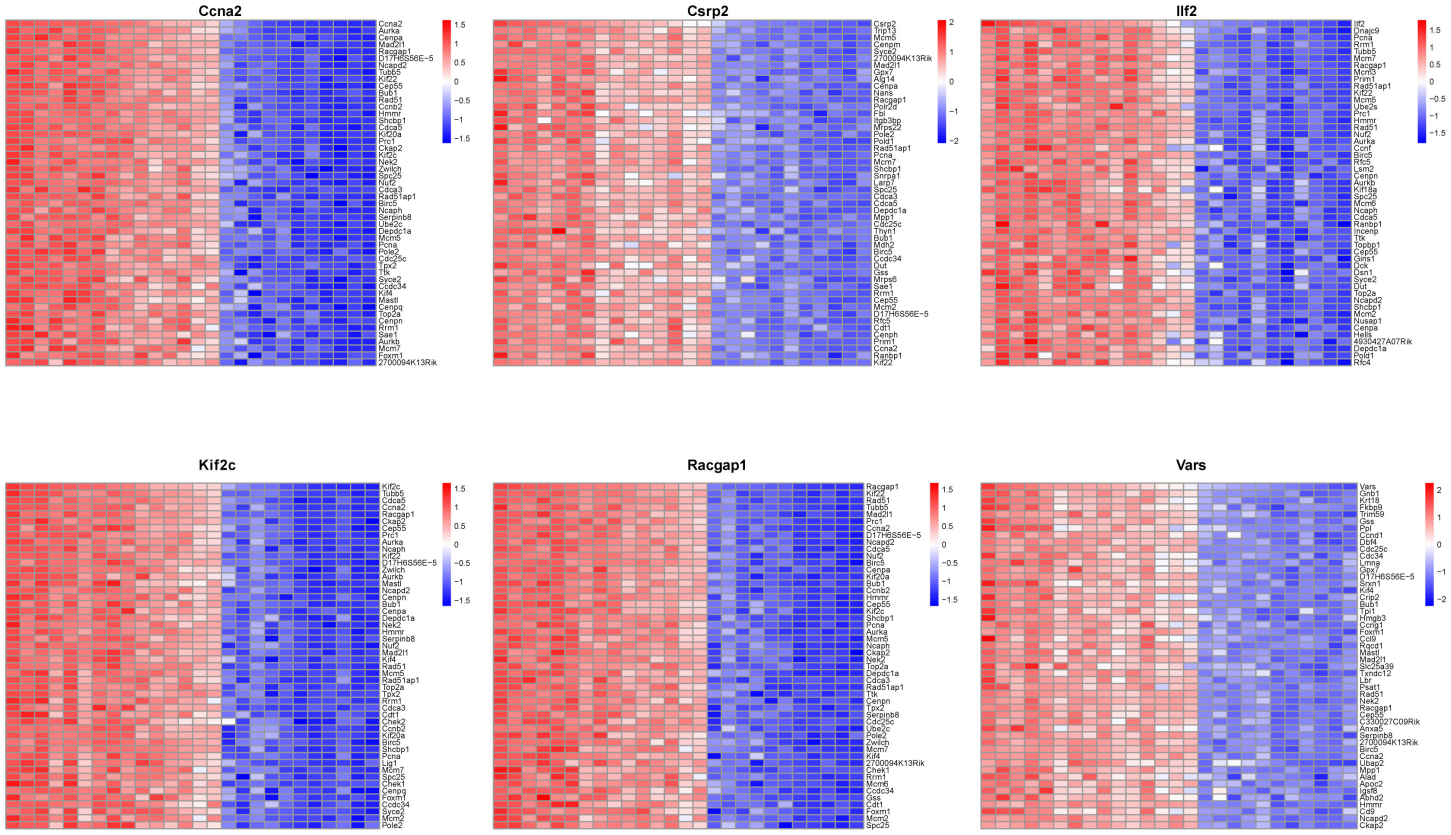
Co-expression network analysis of core genes. A heatmap illustrates co-expression patterns of the top 50 positively correlated genes for each of the 6 core genes, highlighting their interaction network.

Analysis of relationships between core lactylation modification genes and liver regeneration - related genes showed that Ccna2 was correlated with cell cycle - related genes like Aurk and Cenpa, and so on for other genes (**Figure 9**). This implies that these core genes may participate in liver regeneration by regulating cell - cycle - related genes.

### 3.8 Single gene GSEA analysis

**Figure 10:**
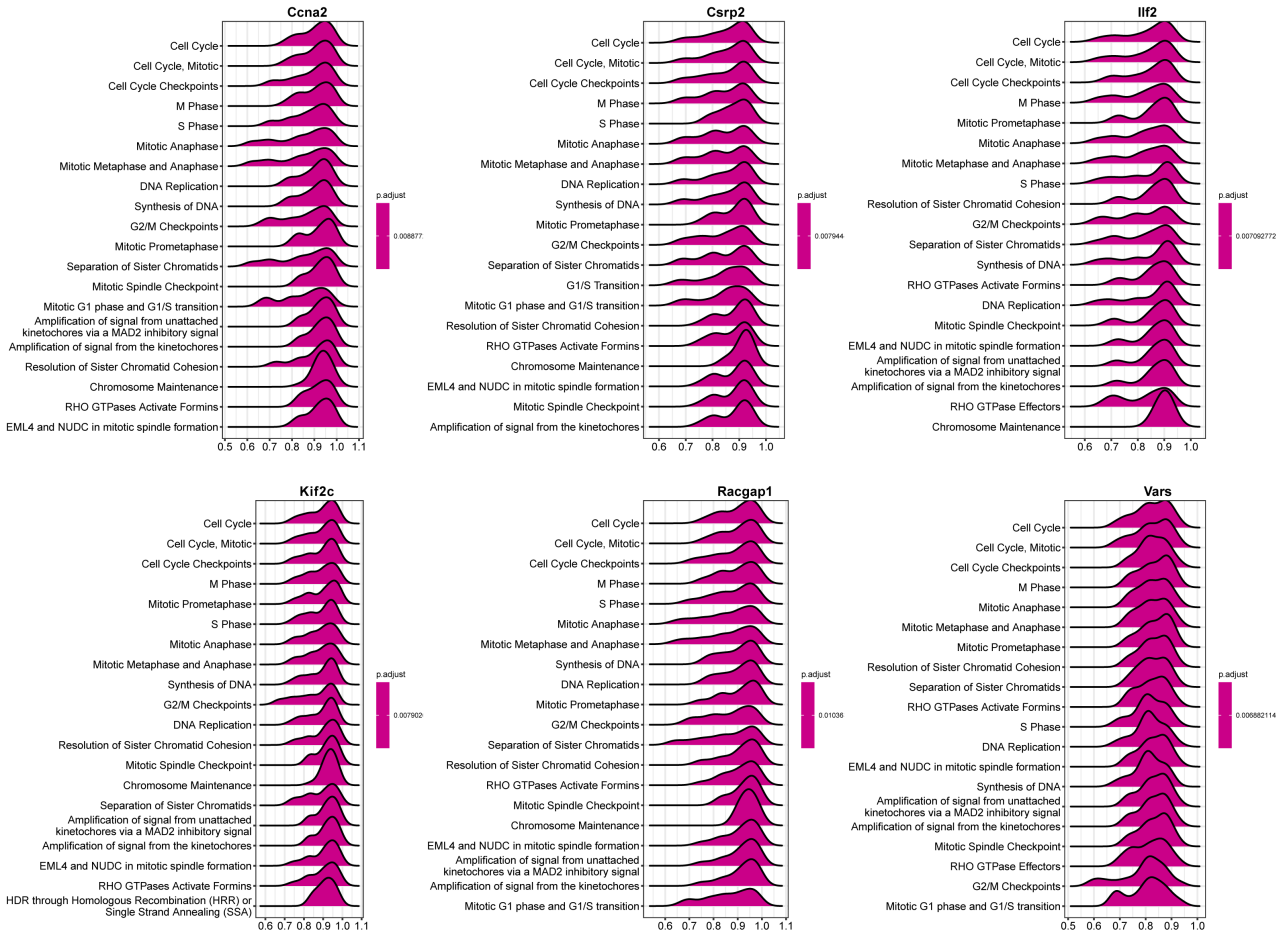
GSEA of core genes. The top 20 reactome pathways enriched for each core gene are shown, with positive enrichment scores indicating upregulation and negative scores indicating downregulation of gene sets.

Single gene GSEA of the six core genes indicated their participation in cell - cycle - related pathways (**Figure 10**). Ccna2 was associated with cell cycle checkpoints, Csrp2 and Ilf2 with the cell cycle and mitosis prophase, etc., suggesting their role in modulating liver regeneration through cell proliferation and division.

### 3.9 Construction of the miRNA-lactylation modification core gene-transcription factor network`

**Figure 11:**
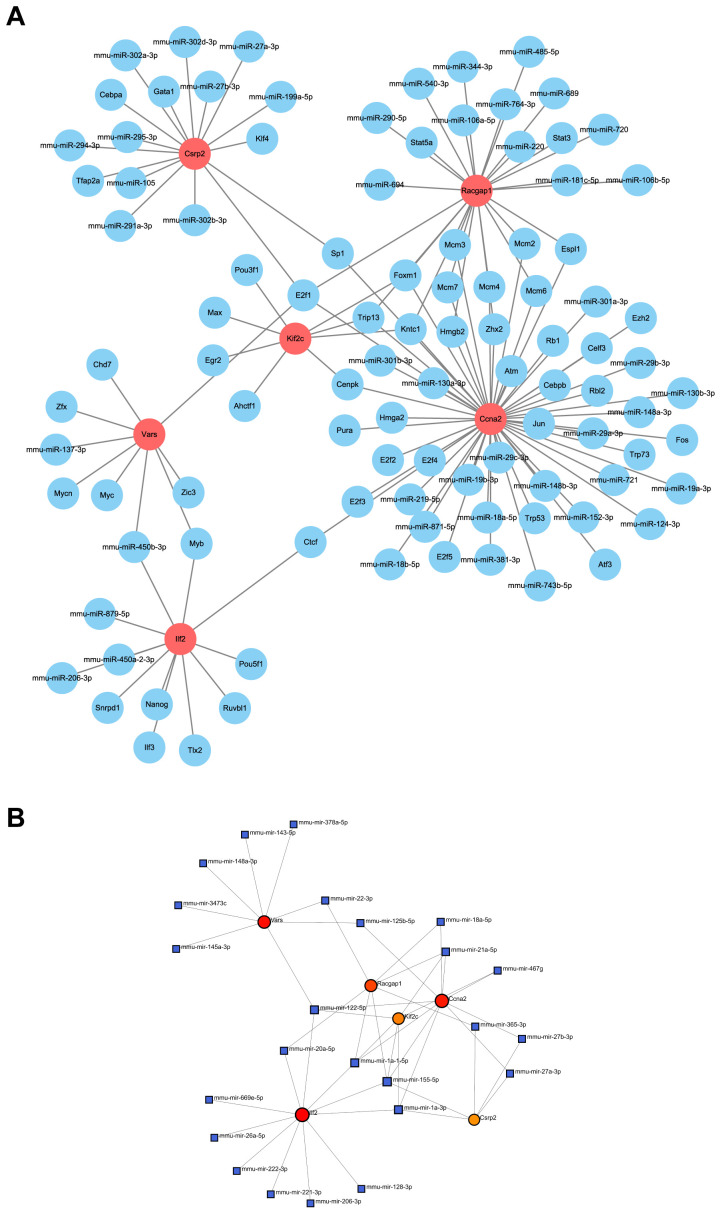
Regulatory network of core genes. **(A)** Interaction network displays the relationship between core genes and upstream miRNAs and transcription factors. **(B)** miRNA regulatory network, as identified by NetworkAnalyst, shows key regulatory elements for core genes.

A miRNA-lactylation modification core gene-transcription factor regulatory network was constructed, with 107 nodes and 123 edges. Mmu - mir - 450b - 3p regulated the most genes, and E2f1 was an important transcription factor (**Figures 11A, B**). Ccna2, as a core gene, was regulated by nine miRNAs and could regulate 32 transcription factors, highlighting its significance in liver regeneration.

### 3.10 Expression and survival analysis of core lactylation modification genes in liver cancer

**Figure 12:**
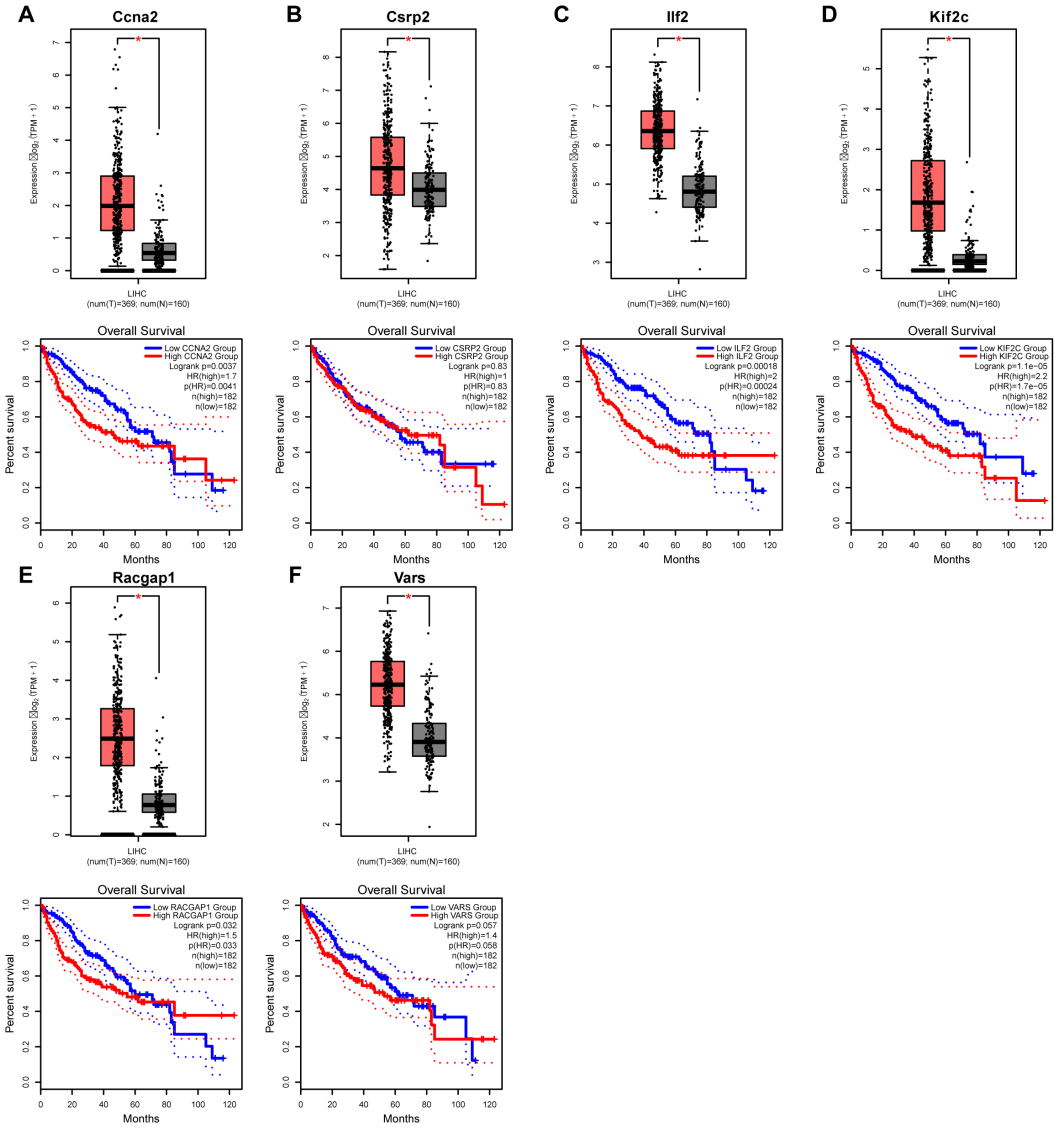
Expression and prognostic analysis of core genes in liver cancer. **(A–E)** Kaplan–Meier survival curves illustrate the relationship between the expression levels of six core genes and overall survival in patients with hepatocellular carcinoma, demonstrating their prognostic significance.

Database analysis showed that the expressions of the six core genes in liver cancer tissues were significantly higher than in normal tissues (P < 0.05) (**Figures 12A–E**). Survival analysis indicated that high expression of CCNA2, CSRP2, ILF2, KIF2C, RACGAP1, and VARS was associated with reduced overall survival rates, especially Kif2c with a very low P-value.

### 3.11 Validation of core lactylation genes in hepatocellular carcinoma by qPCR

**Figure 13:**
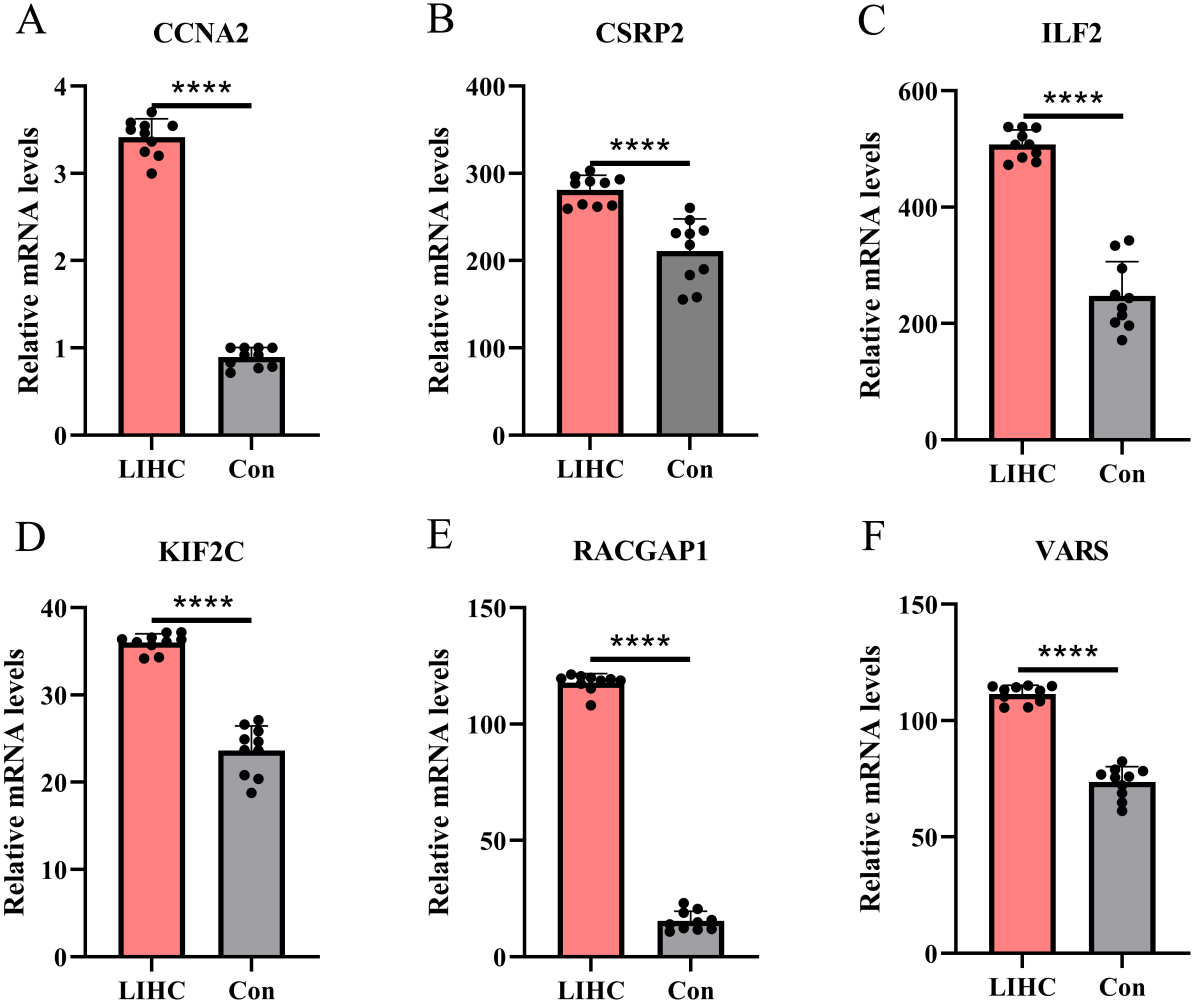
qqPCR-based expression profiling of core genes in LIHC. **(A–E)** qPCR validation reveals significant up - regulation of CCNA2, CSRP2, etc. in liver hepatocellular carcinoma tissues compared to controls (****p < 0.0001).

qPCR validation in normal liver and HCC tissues showed that CCNA2, CSRP2, ILF2, KIF2C, RACGAP1, and VARS were significantly upregulated in the HCC group (all p-values < 0.0001) (**Figure 13**), providing more evidence for their role in liver cancer progression and potential as HCC biomarkers.

### 3.12 Western blot analysis

**Figure 14:**
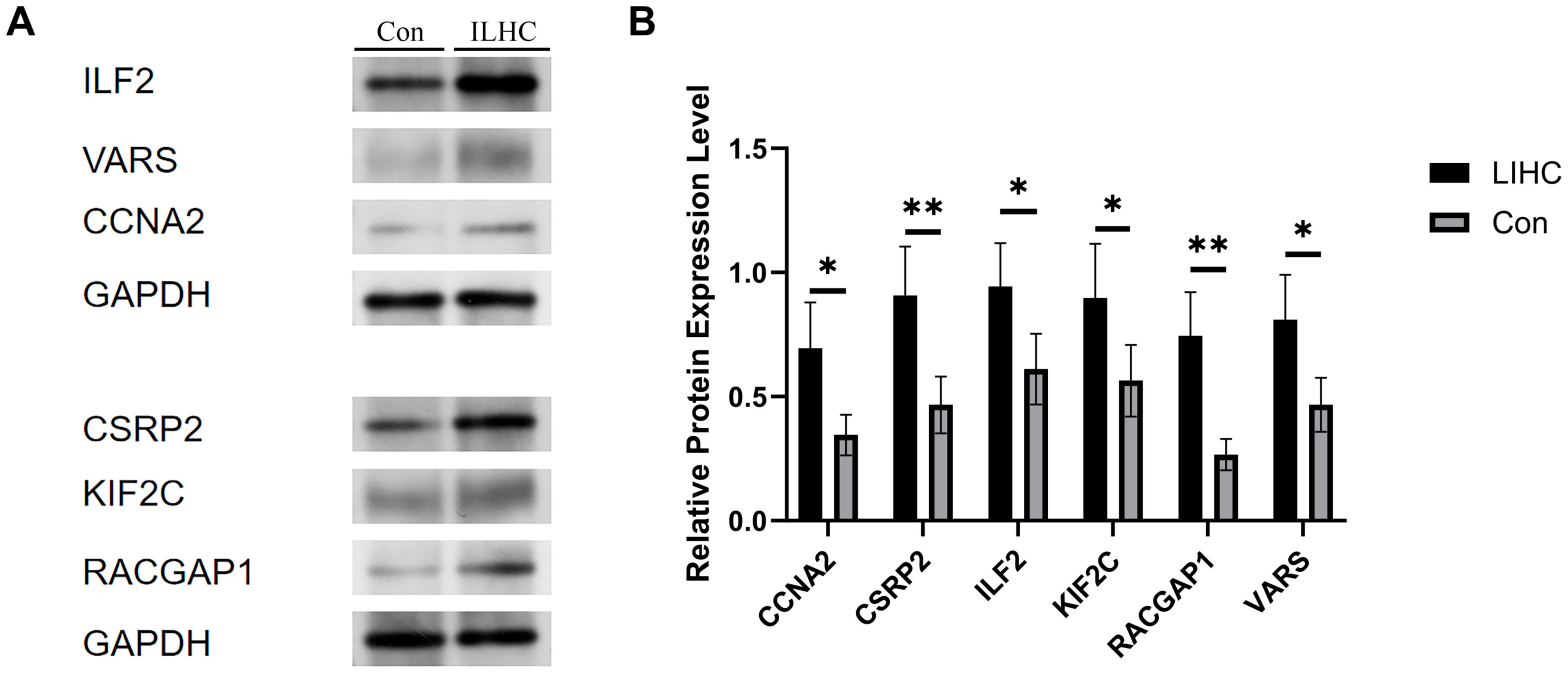
Expression levels of the core gene in normal and tumor liver tissues. **(A)** This figure presents the comparative expression levels of the identified core gene between normal liver tissues and liver hepatocellular carcinoma (LIHC) samples. The bar chart demonstrates a markedly higher expression of the core gene in tumor tissues. **(B)** Statistical analysis reveals that this upregulation is significant, indicating that the core gene may play a critical role in the tumorigenesis and progression of hepatocellular carcinoma. Error bars represent standard deviation across biological replicates (p < 0.05, *p < 0.01).

Western blotting revealed significantly elevated levels of CCNA2, CSRP2, ILF2, KIF2C, RACGAP1, and VARS proteins in HCC tissues compared to adjacent non-tumor tissues (**Figure 14**). Consistently more intense immunoreactive bands at the expected molecular weights confirmed this tumor-specific overexpression across replicates. Densitometric quantification demonstrated statistically significant upregulation (p<0.05) for all target proteins in malignant specimens. Despite the modest, single-center sample size (10 paired samples), both qPCR and Western blot demonstrate consistent upregulation of all six genes in tumors versus adjacent tissues (p < 0.0001), supporting the robustness of the signature. We explicitly acknowledge potential selection bias and limited etiologic/ethnic diversity, and therefore frame these findings as confirmatory; a multi-center expansion with larger, prospectively collected cohorts is outlined in Discussion.

## 4 Discussion

This study systematically integrated three independent liver regeneration-related datasets (GSE20426, GSE70593, and GSE4528) and, for the first time, clearly identified key genes co-regulated by lactylation modifications and liver regeneration. Additionally, we further explored the potential roles and mechanisms of these genes in the occurrence, progression, and metastasis of HCC. Both liver regeneration and HCC involve rapid hepatocyte proliferation; however, there are substantial differences in their regulatory mechanisms and biological significance (30, 31). Liver regeneration represents a strictly regulated physiological proliferative response following hepatic injury, aiming at repairing liver tissue and restoring liver function (32). Conversely, hepatocellular carcinoma is characterized by uncontrolled, pathological proliferation due to dysregulated cell-cycle progression, leading to limitless cancer cell proliferation, invasion, and metastasis (33). Lactylation, as a novel epigenetic modification, has increasingly attracted attention in recent years and is considered closely associated with tumor metabolic reprogramming and remodeling of the tumor microenvironment (6, 34, 35). By conducting bioinformatic analyses of gene interactions between liver regeneration and lactylation modification, this study identified a set of potential marker genes significantly related to hepatocellular carcinoma.

In comparison to previous research on lactylation and HCC, this study significantly extends our understanding by integrating lactylation modification profiles with liver regeneration-associated gene expression data. While earlier studies have established the role of lactylation in metabolic reprogramming and tumor microenvironment modulation, the specific mechanisms through which lactylation influences hepatocyte fate decisions in both physiological and pathological contexts remain poorly defined. This study provides the first comprehensive analysis of the lactylation-driven regulatory network in HCC, revealing a set of six core genes—Ccna2, Csrp2, Ilf2, Kif2c, Racgap1, and Vars—that are uniquely co-regulated by lactylation modifications. By combining multi-omics approaches and machine learning algorithms, we identify lactylation as a key metabolic-epigenetic nexus that connects liver regeneration pathways to oncogenesis, offering novel predictive biomarkers for HCC diagnosis and prognosis. Notably, the identification of Csrp2 as a diagnostic marker with superior efficacy compared to conventional biomarkers such as AFP provides a compelling argument for its clinical application in early HCC detection. Furthermore, the demonstrated correlation between lactylation-associated gene expression and immune microenvironment remodeling adds an innovative layer to our understanding of HCC immune evasion, particularly with respect to resistance to immunotherapies targeting PD-1.

It is important to note that interpreting lactylation as a bridge between regeneration and tumorigenesis is intellectually appealing, but carries a risk of overstatement without direct biochemical evidence. The current analysis primarily infers lactylation effects based on correlated transcriptomic data and previously published literature, and lacks direct histone lactylation profiling of the human HCC samples analyzed in this study. This represents a critical gap, as the core hypothesis relies on lactylation being an epigenetic driver of the identified transcriptional programs—confirmation of this claim would require complementary approaches such as chromatin immunoprecipitation sequencing (ChIP-seq) to detect lactylated histone residues at target gene loci, or mass spectrometry-based lactylome analysis to map global lactylation modifications. Until such direct validation is conducted, the mechanistic links proposed herein remain associative rather than definitive.

To ensure data rigor and reliability, boxplots generated before and after standardizing liver regeneration data demonstrated effective correction of batch effects, confirming the reliability of integrating cross-study data. This strategy not only expanded the sample size but also improved reproducibility in the screening of critical genes, aligning with the methodology proposed by Rho et al. in their multi-omics integration approach for liver regeneration studies (36). Building upon this foundation, the current study further focused on the functions of several key genes and their potential mechanistic roles in HCC.

Differential expression analysis of liver regeneration genes identified 470 upregulated and 323 downregulated genes, which were significantly enriched in nuclear division and cell-cycle pathways, confirming the active proliferation characteristic of liver regeneration. Particular attention was given to genes overlapping between lactylation modification and liver regeneration, revealing enrichment in DNA replication and genome stability pathways. This finding, for the first time, suggests a connection between lactate metabolism and hepatocyte proliferation, thereby extending previous findings by Gao et al. and Wang et al., who demonstrated the involvement of lactylation in metabolic reprogramming and its impact on the tumor microenvironment (37, 38). Furthermore, our enrichment analyses indicate that lactylation-modulated genes are implicated not only in cell cycle and DNA repair but also in immune modulation and metabolic signaling pathways. This suggests a broader functional convergence with established oncogenic cascades in HCC. For instance, the Wnt/β-catenin pathway—a key driver of HCC—is known to promote aerobic glycolysis and lactate production, which in turn may fuel lactylation modifications (20, 21). Similarly, TGF-β signaling, which plays dual roles in liver regeneration and carcinogenesis, has been shown to interact with lactylation-driven immune suppression via regulatory T cells (19, 22). Additionally, JAK/STAT signaling, frequently hyperactivated in HCC, may interface with lactylation-mediated metabolic reprogramming to foster an immunosuppressive microenvironment (23). By intersecting lactylation-related genes with these canonical pathways, our study provides a more integrated view of how metabolic reprogramming epigenetically regulates HCC progression, bridging the gap between lactylation modifications and broader oncogenic network dysregulation.

The core highlight of this study is the precise identification of six key lactylation-related genes (Ccna2, Csrp2, Ilf2, Kif2c, Racgap1, and Vars) using machine learning methods, specifically LASSO and SVM-RFE (39, 40). LASSO regression compresses high-dimensional data noise via a penalty function (41), while SVM-RFE enhances classifier specificity by recursively eliminating weakly correlated variables (42). The combined use of these two methods overcomes the high false-positive rate associated with traditional single-modal analyses, accurately pinpointing sensitive modification nodes.

Through correlation analysis between these genes and immune cells, our research also uncovered significant associations between the core lactylation-related genes and the remodeling of the hepatic immune microenvironment. Taking Ccna2 as an example, its expression positively correlates with pro-inflammatory M1 macrophages but negatively correlates with memory CD8+ T cells. Similarly, Aiello’s study demonstrated that increased CcnA2 expression could reshape tumor-associated macrophages to promote tumor proliferation (43). Such immune microenvironment remodeling may be a crucial reason for resistance to anti-PD-1 immunotherapy in HCC patients (44, 45), suggesting that lactylation-related genes might facilitate immune escape in HCC cells by altering the tumor immune microenvironment (11, 38, 46).

Gene co-expression networks from our study revealed a strong association of Ccna2 with cell cycle proteins such as Aurkb and Cenpa, confirming the regulatory role of lactylation in mitotic fidelity. The connection between Racgap1 and Kif22 indicates lactylation’s potential coordination of spindle assembly and chromosome segregation, defects in which may lead to aneuploidy accumulation, directly associated with HCC pathogenesis. These findings align with Pan et al.’s reports of lactylation promoting chromosomal instability in HCC (47); however, our study uniquely identifies this mechanism as potentially stemming from abnormal activation of physiological regeneration pathways.

GSEA of core genes showed that Ccna2 is enriched in mitotic checkpoint pathways (e.g., PLK1 pathway), while Kif2c is primarily associated with M-phase regulation, consistent with their respective roles in chromosome segregation. Notably, Racgap1 enrichment in S-phase pathways complements known DNA replication stress response mechanisms, suggesting lactylation may regulate cell cycle phase transitions across a temporal dimension. This finding provides a spatiotemporal coupling framework linking metabolic dynamics with epigenetic programming, further enriching Huang et al.’s regulatory model and offering novel targets for cell-cycle synchronization strategies in HCC treatment (48).

The miRNA-lactylation gene-transcription factor network constructed in our study reveals the depth of epigenetic regulation in lactylated genes. For instance, the core gene Ccna2 is regulated by nine miRNAs (e.g., miR-449c-5p) and activates 32 transcription factors, including E2F1, forming a cascade amplification effect. This aligns with the dual role of E2F1 in both tissue regeneration and carcinogenesis (49, 50), suggesting that lactylation modification may amplify E2F1’s pro-proliferative signaling through an epigenetic “driver” mechanism. Notably, the broad-spectrum regulatory capacity of mmu-mir-450 b-3p (targeting Vars and Ilf2) implies its potential role as an upstream coordinator in the lactylation network. These findings provide molecular validation for the “metabolic-epigenetic axis” theory proposed by Zheng and Zhang et al., while suggesting that targeting key miRNAs could systematically modulate the lactylation modification network (51, 52).

Our multidimensional validation integrating clinical correlation and translational potential demonstrated that the six core genes identified in this study exhibited significantly higher expression levels in HCC tissues compared to normal hepatic tissues (p < 0.05). Notably, the elevated expression of Kif2c showed a significant association with poorer patient prognosis, highlighting its potential critical role in HCC malignant progression. Previous studies have revealed that Kif2c promotes tumor invasion and metastasis by inducing chromosomal instability through interference with chromosome segregation processes (53, 54). This finding aligns with the theory proposed by Yao et al. that epigenetic reprogramming drives tumor clonal selection (55), wherein metabolic abnormalities in tumors may induce genomic instability to facilitate clonal selection and evolution. Our database analyses further corroborate this potential mechanism, demonstrating that Kif2c overexpression enhances HCC cell proliferation and survival advantages, ultimately impacting clinical outcomes. First, targeting lactylation directly through inhibition of lactate metabolism enzymes (e.g., LDHA) or lactate transporters (MCT1/4) to reduce intratumoral lactate levels and dampen the aberrant activation of Ccna2, Kif2c, and other core genes, particularly in patients with high glycolytic phenotypes (56); second, exploring synthetic lethal approaches by combining Kif2c-targeted therapy with microtubule-disrupting agents such as paclitaxel, or Racgap1 inhibition with Aurora kinase inhibitors to exploit cell cycle vulnerabilities; third, integrating Csrp2 as a complementary serum biomarker into existing HCC surveillance algorithms alongside AFP and PIVKA-II, especially for AFP-negative cases, and developing multimodal AI-driven risk stratification models that incorporate imaging features and our gene signature to improve early detection; and finally, combining lactylation modulators with immune checkpoint inhibitors to reverse the immunosuppressive microenvironment, given the observed correlations between Ccna2 expression, M1 macrophage infiltration, and reduced CD8+ memory T cell presence (56).

Our qPCR validation further confirmed the overexpression patterns of six candidate genes in clinical HCC specimens, with all genes demonstrating significantly elevated expression levels in HCC tissues compared to normal counterparts. Western blot analysis substantiated these findings at the protein level, revealing significantly elevated expression of CCNA2, CSRP2, ILF2, KIF2C, RACGAP1, and VARS in HCC tissues versus adjacent non-tumor tissues. Densitometric quantification confirmed statistically significant upregulation (p<0.05) for all six proteins, aligning with transcriptomic data. This spatially resolved protein overexpression within the HCC microenvironment reinforces their constitutive activation in malignancy. This experimental verification strengthens the conclusions derived from database analyses and expands their clinical applicability and translational potential. Notably, the Csrp2 gene exhibited remarkable diagnostic value, demonstrating a diagnostic efficacy (AUC > 0.8) slightly lower than the conventional HCC biomarker alpha-fetoprotein (AFP, typically AUC range: 0.78–0.979) (57, 58). These findings suggest that Csrp2 may serve as a novel and efficient biomarker for early HCC detection with promising clinical translation prospects. It is noteworthy, however, that the superior diagnostic performance of Csrp2 (AUC > 0.8) observed in this study was derived from tissue-based expression analysis, which differs fundamentally from serum-based biomarkers currently used in clinical surveillance, such as AFP and des-γ-carboxy prothrombin (DCP). While tissue-based AUC values provide valuable mechanistic insight, they may not directly translate to non-invasive diagnostic settings. Therefore, the potential utility of Csrp2 should be viewed as complementary to existing surveillance strategies—primarily ultrasound combined with serum AFP—rather than as an immediate replacement. Future studies are warranted to validate Csrp2 expression in peripheral blood and to assess its performance in longitudinal surveillance cohorts before any clinical integration can be considered (59, 60). Furthermore, considering recent advancements in lactate metabolism detection technologies, particularly the lactylation profiling technique developed by Wu et al., a Csrp2-centred peripheral blood detection method holds potential for developing non-invasive early screening protocols for HCC. This approach could provide robust technical support for early diagnosis and precise medical interventions in clinical HCC management (61).

Based on our aforementioned research and relevant literature, we have identified that these genes (Ccna2, Csrp2, Ilf2, Kif2c, Racgap1, Vars) may play pivotal roles in the initiation and progression of HCC. Specifically, Ccna2, Kif2c, and Racgap1 primarily promote tumor cell proliferation and division by regulating the cell cycle and mitotic processes. In contrast, Csrp2, Ilf2, and Vars enhance the invasive potential and survival capabilities of HCC cells through their influence on cell migration, transcriptional regulation, and metabolic activities. Consequently, these genes not only occupy critical positions in the molecular mechanisms underlying HCC but also hold promise as potential molecular biomarkers and therapeutic targets, offering novel research directions and clinical prospects for early diagnosis, targeted therapy, and prognostic evaluation of HCC.

For instance, mechanistically, the Ccna2 gene—a key regulator of the cell cycle—may directly drive hepatocarcinogenesis and rapid progression via aberrant overexpression that disrupts cell cycle checkpoints in HCC (62, 63). This observation aligns with the findings of Gao et al., who demonstrated that lactylation modification activates DNA replication-associated pathways to accelerate cellular proliferation, a conclusion highly consistent with our functional predictions for Ccna2 in this study (64).

While this alignment supports the hypothesis that lactylation drives Ccna2-dependent cell cycle dysregulation, it is important to emphasize that we have not directly demonstrated lactylation of Ccna2 itself or lactylation of histones at the Ccna2 locus in HCC cells. Future studies employing ChIP-seq with antibodies specific to lactylated histones (e.g., H3K18la, H3K27la) or mass spectrometry to detect lactylation on Ccna2-interacting proteins would be essential to confirm this mechanistic link.

The Racgap1 gene encodes a Rho family GTPase-activating protein predominantly involved in cytokinesis regulation to ensure orderly cell division. Elevated Racgap1 expression has been shown to induce abnormal cytokinesis, promoting the accumulation and expansion of aneuploid cells that accelerate malignant tumor progression. These findings corroborate reports by Pan et al. on lactylation-enhanced chromosomal instability in tumors (36). Our study further hypothesizes that hyperactive lactylation modification may drive hepatocytes to shift from reparative proliferation to pathological proliferation patterns, potentially representing a critical mechanism in HCC pathogenesis (65, 66).

The Kif2c gene is significantly upregulated in HCC tissues and exhibits a strong correlation with poor patient survival rates, highlighting its substantial potential as a clinical prognostic biomarker. Additionally, our study revealed a distinct dose-dependent relationship between Kif2c expression levels and intracellular lactate concentration. This finding aligns closely with the theory proposed by Zhou et al., which posits that metabolic disorder-mediated epigenetic modifications drive HCC progression (67). Further functional investigations demonstrated that Kif2c inhibition markedly enhances the sensitivity of HCC cells to glycolysis inhibitors, suggesting that Kif2c may serve as a critical node within a metabolic-epigenetic crosstalk regulatory network.

Ilf2 and Vars are identified in this study, for the first time, as core lactylation-associated genes closely related to HCC. Although the specific molecular mechanisms of these two genes have not yet been fully elucidated, existing evidence suggests that Ilf2 may enhance the proliferative capacity of HCC cells by regulating cell cycle-related gene networks, whereas Vars may affect cancer cell survival and proliferation through lactylation-mediated translational regulation. Further investigation into these two genes will contribute to a more comprehensive understanding of the mechanisms underlying HCC pathogenesis and progression.

Particularly noteworthy is the Csrp2 gene. Csrp2 not only exhibits significantly higher expression levels in tumor tissues compared to normal tissues but also demonstrates superior diagnostic performance compared to AFP, a traditional biomarker for HCC. It is speculated that Csrp2 may promote tumor cell migration and invasion by influencing cytoskeletal stability and activating signaling pathways, aligning with the mechanisms reported by Gu et al., which indicate lactylation-mediated modulation of structural proteins influences tumor metastasis (68). Therefore, Csrp2 represents a promising novel target for early diagnosis and treatment of HCC, possessing substantial potential for clinical translation.

Based on these findings, we further explored the dynamic changes in lactylation modifications during HCC initiation and progression. Traditional HCC studies often utilize static tumor-versus-normal tissue comparison models, making it challenging to capture dynamic epigenetic modifications (68). In this study, we innovatively introduced the PHx liver regeneration model as a platform for studying carcinogenic evolution, thereby revealing critical thresholds at which lactylation modification networks shift from physiological repair processes to pathological malignancy. The continued activation of lactylation-associated genes in the DNA replication pathway closely aligns with the clinical progression from liver cirrhosis to hepatocellular carcinoma.

The limitations and summary of this study are as follows. Several aspects require further investigation: The PHx model does not adequately replicate the genetic heterogeneity observed in human HCC; therefore, complementary cirrhosis progression models and organoid validations are necessary. Human hepatocellular carcinoma (HCC) is characterized by significant genetic and tumor microenvironmental heterogeneity, which complicates direct translation from murine models. In this study, we used murine liver regeneration models, specifically partial hepatectomy, to investigate molecular mechanisms underlying liver regeneration and HCC. While these models offer valuable insights due to the genetic and molecular similarities between murine and human livers, they present limitations in fully capturing the complexity of human HCC. Human liver regeneration data, crucial for more direct insights, are difficult to obtain due to ethical constraints and the lack of large, accessible datasets. As a result, murine models, though informative, cannot completely replicate the diversity of human conditions. Future studies should incorporate human-derived datasets and clinical validation to better connect murine models with human liver biology, providing more accurate and translatable findings in the context of HCC development and progression. Furthermore, bulk sequencing-based immune infiltration analyses may obscure modification specificity in hepatic macrophages such as Kupffer cells; employing spatial transcriptomics could enhance analytical resolution. Although the sample size for the initial clinical validation (10 normal vs 10 HCC tissues) is modest, we employed rigorous bioinformatics preprocessing to mitigate this limitation. Specifically, the integration of multiple genomic datasets (GSE20426, GSE70593, GSE4528) followed by batch effect removal using the ‘sva’ package and quantile normalization using the ‘preprocessCore’ package is a recognized approach to enhance the robustness and reliability of findings derived from smaller cohorts (21, 23). This strategy strengthens the foundational bioinformatics discovery phase. Nevertheless, the conclusions drawn from the clinical validation cohort, while statistically significant and experimentally corroborated by Western blot, would benefit from future validation in larger, independent clinical cohorts to confirm the generalizability and translational potential of our identified lactylation gene signature. Fourth, our prognostic and diagnostic analyses have certain constraints.

Survival analysis charts derived from online databases are indeed commonly used in many studies, and they can provide valuable insights, especially when detailed clinical data is not readily available. These charts allow for a broad analysis of gene expression patterns and their associations with survival outcomes, offering an initial understanding of potential However, it is essential to recognize that such analyses have limitations, particularly when clinical covariates are not accounted for. The use of these charts should be viewed as preliminary, the future studies with more comprehensive clinical data would be necessary to strengthen the conclusions and confirm the findings. The prognostic value of the core lactylation genes was evaluated using univariate Kaplan-Meier survival analysis. While this provides robust evidence of association, the lack of accessible detailed clinical data (such as TNM stage, Child-Pugh score, and treatment history) in the public repositories precluded us from performing multivariate Cox regression analysis to determine the independent prognostic power of our gene signature (26, 69). Similarly, a direct comparison of the diagnostic accuracy of tissue-based Csrp2 expression with serum AFP levels was not feasible due to the inherent differences in sample types and cohort sources between gene expression datasets and serum biomarker datasets (28, 29). Future studies with prospectively collected cohorts containing matched tissue, blood samples, and comprehensive clinical information are essential to validate the independent prognostic value and diagnostic efficacy of our identified lactylation-associated genes (70, 71).

The innovative aspect of this study lies in the pioneering use of a liver regeneration model to reveal the dynamic transformation of lactylation modifications from physiological repair processes to pathological proliferation, offering a novel perspective for understanding HCC development. Future studies should employ specific lactylation enzyme probes and single-cell omics technologies to more accurately elucidate the dynamic changes and underlying mechanisms of lactylation modifications in HCC, thus providing robust theoretical and practical foundations for diagnosis and treatment strategies.

## 5 Conclusion

Based on the dynamic regulatory network of liver regeneration and lactylation modification, this study successfully identified six core genes, namely Ccna2, Csrp2, Ilf2, Kif2c, Racgap1, and Vars, as potential therapeutic targets and biological biomarkers for HCC. These genes mediate the epigenetic transition from physiological liver regeneration to pathological carcinogenesis by orchestrating critical mechanisms, including cell cycle checkpoints, chromosomal stability, and immune microenvironment remodeling. Clinical translational analysis revealed that Csrp2 demonstrates superior diagnostic efficacy (AUC >0.8) in HCC tissues compared to conventional biomarker AFP, with its peripheral blood detection technology showing promise for non-invasive early screening of liver cancer. Meanwhile, elevated Kif2c expression exhibits a strong correlation with poor patient prognosis, serving as a valuable stratification indicator for personalized treatment. Significantly, the potential association between lactylation-modified genes and immune checkpoint inhibitor resistance (e.g., the Ccna2-M1 macrophage axis) uncovered in this study provides novel insights for developing combined “metabolism-immunity” therapeutic strategies. These findings not only reinterpret hepatocarcinogenesis through the lens of metabolic-epigenetic coupling, but also establish a molecular toolkit with both diagnostic sensitivity and therapeutic targeting potential for constructing clinical precision medicine systems.

## Data Availability

The datasets presented in this study can be found in online repositories. The names of the repository/repositories and accession number(s) can be found in the article/supplementary material.
